# Testosterone Replacement Therapy: Should It Be Performed in Erectile Dysfunction?

**DOI:** 10.5812/numonthly.11523

**Published:** 2013-07-29

**Authors:** Orcun Celik, Selcuk Yücel

**Affiliations:** 1 Clinic of Urology, Kemalpasa State Hospital, Kemalpasa, Izmir, Turkey; 2 Department of Urology, Medical Faculty, Akdeniz University, Antalya, Turkey

**Keywords:** Testosterone, Erectile Dysfunction, Therapeutics

## Abstract

The classical etiology of erectile dysfunction (ED) comprises aging and vascular, neurogenic, psychological and hormonal components. Recent studies have shown that ED can be the forerunner of serious cardiovascular disturbances. It has also been reported that peripheral neuropathy and microvascular injuries caused by pathophysiological changes in patients with diabetes and obesity lead to ED in a significant number of such cases. These patients develop clinically significant ED and comprise a significant portion of the patient group which do not respond to PDE-5 inhibitors. Testosterone has been shown to increase the expression of PDE-5. This function of testosterone supports its effect on the regulation of erection and increasing the sexual libido. In view of the complexity of ED, as well as the effect of testosterone on erection, it is concluded that PDE-5 inhibitors in combination with testosterone replacement would be a better therapy alternative in the management of erectile dysfunction in hypogonadal patients.

## 1. Introduction

A study reported that there were about 152 million men with complaints of erectile problems in the whole world in 1995, and this number would rise to approximately 322 million in 2025 (1). Particularly in the early 1980s, significant advances in the knowledge and comprehension of erectile physiology were made; new knowledge regarding the importance of organic causes has led to the change of prevailing perception that most EDs have a psychogenic origin (2). Since ED is a disease of the aging, it is quite difficult to determine an isolated single factor in its etiology, because in aged individuals, ED can be caused by various factors, such as systemic diseases including diabetes mellitus (DM), renal insufficiency and cardiovascular diseases, hormonal changes, chronic use of medications, surgical interventions and aging of tissues. Recent studies have shown that testosterone (T) deficiency can lead to diseases with potential mortality such as metabolic syndrome, DM, osteoporosis, bone fractures and coronary artery disease. Although the role of hormones in ED has not been fully clarified, some indicative data have been obtained. Hormones that may be possibly related to ED are androgens (testosterone = T, dihydrotestosterone = DHT, androstenedione, dehydroepiandrosterone = DHEA and dehydroepiandrosterone-sulphate = DHEA-SO4), estrogens (in particularly, estradiol = E2), insulin (cause of DM and consequently, an indirect cause of ED), thyroid hormones, prolactin (PRL), melatonin, leptin and growth hormone (GH). It has been demonstrated that hormones are responsible for about 5% of ED cases with organic causes. In particular a serum T level of < 300 ng/dL is found in 10-20% of ED patients (3, 4).

## 2. Physiology of Testosterone

Testosterone is derived from pregnenolone in Leydig cells. The daily release of T in male is 5 mg, and its secretion is pulsatile. The release of T shows a diurnal pattern; the secretion attains a peak in the early morning hours and is lowest in the evening and night hours. Testosterone can be converted by the 5-alpha-reductase enzyme to DHT in androgen target cells. Both hormones bind to the same high-affinity receptor and then as a hormone-receptor complex, pass to the cell nucleus to show their biological activity. Testosterone can be converted by the aromatase enzyme to estrogens, whereas DHT cannot. Like other steroid hormones, after binding to high-affinity receptors, the androgens and estrogens show their effects at cellular level. The androgen receptors are found in relatively high concentrations in androgen target tissues. In the testes, the androgen receptors are located in both the Sertoli and the Leydig cells. In normal males, %2 of T is free and 30% is bound with high affinity to the sex hormone binding globulin (SHBG). The remaining T is bound with lower affinity to albumin and other proteins. The testosterone fractions not bound to SHBG are designated as bioavailable T. Binding proteins regulate the T fractions. Previously, physiological active androgen was considered to be the “free” T (f-T) unbound to protein. However, it has recently been shown that transport of steroid hormones within the cell is much more complicated and that separation of the hormone from the binding protein in the microcirculation is much more rapid than formerly known. Again, recent studies demonstrated that albumin-bound T was found to be bioavailable when transferred to target tissues in organs such as the brain and the liver. The affinity of SHBG for T is more than its affinity to E2, and changes in the SHBG levels are reflected as an increase or a decrease in hormones. In the process of aging, the increases in estrogens, thyroid hormone, and healthy aging reduce the f-T fraction by increasing the plasma SHBG (5). In the male, androgens play a significant role in the physiology of organs such as muscle, central nervous system (CNS) organs, prostate and bone marrow, as well as in the physiology of sexual function. The biological effects of T and its metabolites are classified according to the sites affected. The effects related to the development of the male reproductive system or the secondary sex characteristics are designated as the androgenic effect and the effects that stimulate the growth or trophic effects in somatic tissues are designated as the anabolic effect. The androgens are responsible for prenatal differentiation and development of the male reproductive system. Furthermore, these hormones play a key role in the stimulation and continuation of sexual function in the male. Testosterone is also essential for normal libido, ejaculation and spontaneous erection. Different threshold values varying in individuals with different types of sexual dysfunction have been reported (6). Androgens have a significant role in the expression of neuronal nitric oxide synthase (n-NOS) and phosphodiesterase-5 (PDE-5) genes (7). Recently, experimental animal studies have demonstrated that normal androgen levels are a prerequisite for the appropriate effect of PDE-5 inhibitors, approving the functions of the afore-mentioned androgens. The same studies have also shown that androgen deprivation (surgical or medical) leads to insufficiency of the veno-occlusive mechanism by causing significant structural changes in cavernous bodies (8). Androgens are also effective on serum lipids. When males were compared with premenopausal females, it was found that in general, the plasma levels of high-density lipoprotein (HDL) cholesterol in males were lower and that the plasma levels of triglyceride, low-density lipoprotein (LDL) cholesterol and the very-low-density lipoprotein (VLDL) were higher than those in females. On the other hand, hyperlipidemia is accepted as a risk factor for ED. For this reason, study of the lipid profile is recommended in cases examined for ED (9).

### 2.1. Relationship Between Testosterone and Erectile Dysfunction

Experimental animal studies have demonstrated the relationship between testosterone and ED. Traish et al. obtained very important evidences on the relationship between erectile function and dysfunction and T. In their experimental study on rabbits, the authors histopathologically examined the erectile tissues of male rabbits and evaluated the smooth muscles and connective tissue. Interestingly, they noted a reduction in smooth muscle and an increase of connective tissue in the erectile tissue, along with accumulation of adipocytes in the subtunical region of the corpus cavernosum. Thereupon, they put forth the hypothesis that androgen deprivation caused the stromal progenitor cells to turn into adipocytes. Their evidence-based study showed that the veno-occlusive mechanism, which is one of the most frequent causes of ED, could be a result of this accumulation of fat cells (10, 11). Armağan et al. have already shown that the above-mentioned histopathological changes can be corrected with T replacement (12). Recently, Yıldız et al. reported that T opens the potassium channels of smooth muscles in human penile cavernous bodies, thus providing muscle relaxation and contributing to erection (13). Nitric oxide enables penile erection by mediating the relaxation of the vascular and trabecular smooth muscles in the corpus cavernosum. It has been reported that androgens regulate the expression of nitric oxide isoforms in the corpus cavernosum (14). Experimental animal studies have shown that following castration, there is a significant fall in penile nNOS and endothelial NOS (eNOS) in the western blot and biochemical analyses, and that these biochemical and metabolic changes can be normalized with testosterone therapy (15, 16). The inhibition of the PDE-5 enzyme, which is responsible for the hydrolysis of cGMP in trabecular smooth muscles, increases the cGMP-mediated smooth muscle relaxation, restoring penile erection in cases with ED (14-16). Orchiectomized animals showed failed activity of PDE-5, which was restored with T therapy (14). Experimental animal studies have demonstrated that the deficiency of androgens lead to penile atrophy, changes in the structure of the dorsal nerve, reduction in the content of trabecular smooth muscle, increased deposition of the extracellular matrix, and accumulation of adipocytes in the subtunical region. The regulation of the composition, organization and fibroelastic features of penile cavernosal tissue by androgens is the critical point for veno-occlusion and erectile function. Androgen deficiency breeds the veno-occlusive dysfunction by causing metabolic, structural and functional changes in the corpus cavernosum. Although all these evidences distinctly indicate the role of T in ED, there are still concerns about T therapy and no full consensus on who should receive T replacement therapy has been reached yet.

### 2.2. Testosterone Replacement Therapy (TRT)

In patients for whom testosterone replacement therapy (TRT) has been planned, the complaints and symptoms related to erectile dysfunction can be nonspecific. Although there is no definite evaluation method for the diagnosis, in the first examination, questionnaires such as the IIEF (international index of erectile dysfunction), ADAM (androgen decline in the aging male) and AMS (aging male survey) should be used. Investigation of the serum T level is recommended when there are high symptom scores accompanied by ED, lack of libido, loss of muscle mass, metabolic syndrome and diabetes mellitus type 2 (17). Moreover, the effects of medications (ketoconazole, chemotherapeutics, glucocorticoids, opioids, etc.) used by the patient should also be considered. Testing the early morning total testosterone (TT) in blood is also recommended. A serum TT level of > 346 ng/dL indicates the absence of hypogonadism; when the level is < 231 ng/dL, the TT test should be performed again, along with serum LH, FSH, prolactin and ferritin tests, and when the level is between 231-346 ng/dL, the bioavailable T (bT) should be investigated. In patients with serum TT values of < 231 ng/dL, TRT should be initiated; in patients with values between 231-346 ng/dL, if the bT is low, first the “testosterone therapy test” should be performed and the therapy should either be continued or stopped according to the results of the test (18). Recently, the testosterone therapy test has been recommended for patients whose TT levels are at the limit or low/normal (231-346 ng/dL). In this test, TRT is begun with normal doses; after three months, the patient is evaluated according to his symptoms; if there is a response, the therapy is continued; if there is no response, other factors in the etiology are investigated (19). In their meta-analysis covering (17) studies, Isidori et al. reported that the effect of TRT in ED was directly related to the serum T levels. When compared to placebo, the patients who benefited most from the therapy were those with serum TT levels of < 231 ng/dL; patients with serum TT levels between 231-346 ng/dL benefited moderately, and those with TT levels of > 346 ng/dL had no benefit at all (20). One of the current topics of TRT is the effect of intermittent therapy. In studies in which intermittent therapy is preferred, it has been reported that symptomatic cure continues after the therapy in spite of the low T levels.

Today, there are many T formulations to be used in TRT. The oral forms of T are not highly preferred, due to the fact that they generally attain T levels under the physiological limits and cause liver toxicity. The patch forms of T are of limited use due to their side effects and the frequent need for shaving. Although short-acting mixed T esters cause fluctuations in the serum T levels and therefore not ideal, they are frequently used in many countries due to their low price. Testosterone gel and undecanoat T depot intramuscular (IM) both provide normal serum values. However, with the IM form, a higher level of serum T is always attained. The values formerly determined in the IIEF (international index of erectile function questionnaire) and AMS, as well as all the parameters of metabolic syndrome are better restored with the IM form than with the gel form.

### 2.3. Testosterone Replacement Therapy (TRT) and PDE-5 Inhibitors

The first choice of therapy in ED is PDE-5 inhibitors, but this therapy results in no response in 30-50% of the patients. When TRT is added to the therapy of nonresponders, the outcome is positive (21). Although the protocol of combined therapy with TRT + PDE-5 inhibitors for hypogonadism in ED is still disputable, TRT is recommended for the first 6 months and if there is no response, the addition of PDE-5 inhibitors to the therapy is undertaken (22). In studies on the effect of combined therapy, a definite treatment algorithm could not be determined due to insufficient number of samples, different cut-off values of T, and absence of placebo controls (23-27). In a recent meta-analysis of three randomized controlled studies on hypogonadal patients with ED giving no response to PDE-5 inhibitors, it could not be determined whether oral PDE-5 inhibitors + TRT exerted a significant effect on sexual function, compared to PDE-5 inhibitor therapy alone (28). A recent multicenter, double-blind, placebo-controlled study (TADTEST Study) produced contrasting results. The study included 178 male patients who had formerly not responded to therapy with PDE-5 inhibitors and who had serum T levels of ≤ 400 ng/dL (13.8 nmol/L). All the patients had received oral Tadalafil 10 mg once daily for 4 weeks. The patients not responding to Tadalafil were divided into two groups, with one group additionally receiving placebo and the other group additionally receiving 0.1% testosterone gel.

At the end of 12 weeks, there was no additional effect observed in patients with T levels of 337 ± 14.8 ng/dL (11.7 ± 0.5 nmol/L) receiving Tadalafil + T gel. On the other hand, in hypogonadal ED patients with T levels under the basal values [≤ 300 ng/dL (≤ 10.4 nmol/L)], there was a significant symptomatic restoration in the IIEF scores and the frequency of sexual intercourse, compared to the placebo group (29).

In conclusion, in the therapy of ED, particularly in patients with late-onset hypogonadism, TRT should be used in cases that are unresponsive to PDE-5 inhibitors, which promotes significant symptomatic relief.
